# Population-Modifiable Risk Factors Associated With Childhood Stunting in Sub-Saharan Africa

**DOI:** 10.1001/jamanetworkopen.2023.38321

**Published:** 2023-10-18

**Authors:** Kedir Y. Ahmed, Abel F. Dadi, Felix Akpojene Ogbo, Andrew Page, Kingsley E. Agho, Temesgen Yihunie Akalu, Adhanom Gebreegziabher Baraki, Getayeneh Antehunegn Tesema, Achamyeleh Birhanu Teshale, Tesfa Sewunet Alamneh, Zemenu Tadesse Tessema, Robel Hussen Kabthymer, Koku Sisay Tamirat, Allen G. Ross

**Affiliations:** 1 Rural Health Research Institute, Charles Sturt University, Orange, New South Wales, Australia; 2 Charles Darwin University, Menzies School of Health Research, Northern Territory, Australia; 3 Addis Continental Institute of Public Health, Addis Ababa, Ethiopia; 4 Riverland Academy of Clinical Excellence, Riverland Mallee Coorong Local Health Network, South Australia Health, Government of South Australia, Berri, South Australia, Australia; 5 Translational Health Research Institute, Western Sydney University, Campbelltown Campus, Penrith, New South Wales, Australia; 6 School of Health Sciences, Western Sydney University, Campbelltown Campus, Penrith, New South Wales, Australia; 7 School of Population Health, Faculty of Health Sciences, Curtin University, Bentley, Western Australia, Australia; 8 Geospatial and Tuberculosis Research Team, Telethon Kids Institute, Perth, Western Australia, Australia; 9 Institute of Public Health, College of Medicine and Health Sciences, University of Gondar, Gondar, Ethiopia; 10 School of Rehabilitation Therapy, Queen’s University, Kingston, Ontario, Canada; 11 School of Public Health and Preventive Medicine, Monash University, Melbourne, Victoria, Australia; 12 Population Health Sciences, Bristol Medical School, University of Bristol, Bristol, United Kingdom; 13 Department of Medicine, School of Clinical Sciences, Monash University, Melbourne, Victoria, Australia; 14 Department of Human Nutrition, School of Public Health, Dilla University, Dilla, Ethiopia; 15 School of Rural Health, Monash University, Warragul, Victoria, Australia

## Abstract

**Importance:**

Identifying modifiable risk factors associated with childhood stunting in sub-Saharan Africa (SSA) is imperative for the development of evidence-based interventions and to achieve the Sustainable Development Goals.

**Objective:**

To evaluate key modifiable risk factors associated with childhood stunting in SSA.

**Design, Setting, and Participants:**

This cross-sectional study examined the most recent (2014-2021) Demographic and Health Surveys data for children younger than 5 years from 25 SSA countries.

**Exposures:**

Modifiable risk factors included history of diarrhea within 2 weeks, consumption of dairy products, maternal body mass index, maternal educational level, antenatal care visits, place of birth, wealth index, type of toilet, and type of cooking fuel.

**Main Outcomes and Measures:**

Stunting and severe stunting, measured using the height-for-age *z* score, were the main outcomes. Children who scored below −2.0 SDs or −3.0 SDs were classified as having stunted or severely stunted growth, respectively. Relative risks and 95% CIs were computed using generalized linear latent and mixed models and log-binomial link functions. Population-attributable fractions (PAFs) were calculated using adjusted relative risks and prevalence estimates for key modifiable risk factors.

**Results:**

This study included 145 900 children from 25 SSA countries. The mean (SD) age of the children was 29.4 (17.3) months, and 50.6% were male. The highest PAFs of severe childhood stunting were observed for mothers lacking a formal education (PAF, 21.9%; 95% CI, 19.0%-24.8%), children lacking consumption of dairy products (PAF, 20.8%; 95% CI, 16.8%-24.9%), unclean cooking fuel (PAF, 9.5%; 95% CI, 2.6%-16.3%), home birth (PAF, 8.3%; 95% CI, 6.3%-10.0%), and low-income household (PAF, 5.8%; 95% CI, 3.4%-8.0%). These 5 modifiable risk factors were associated with 51.6% (95% CI, 40.5%-60.9%) of the severe childhood stunting in SSA.

**Conclusions and Relevance:**

This cross-sectional study identified 5 modifiable risk factors that were associated with 51.6% of severe childhood stunting in SSA. These factors should be a priority for policy makers when considering future child health interventions to address chronic malnutrition in SSA.

## Key Points

**Question:**

What are the key modifiable risk factors associated with childhood stunting in sub-Saharan Africa (SSA)?

**Findings:**

In this cross-sectional study of 145 900 children younger than 5 years from 25 SSA countries, mothers’ lack of formal education, children’s lack of sufficient dairy product consumption, unclean cooking fuel, home births, and low-income households were key modifiable risk factors associated with 52% of severe childhood stunting.

**Meaning:**

Addressing these key modifiable risk factors may substantially contribute to reducing severe childhood stunting in SSA populations.

## Teaser

This cross-sectional study examines population-modifiable risk factors associated with childhood stunting and severe stunting in sub-Saharan Africa among children younger than 5 years.

## Introduction

Childhood malnutrition remains a critical public health challenge worldwide, encompassing issues such as stunting, wasting, underweight, and micronutrient deficiencies.^1,2^ Malnutrition has been associated with adverse short- and long-term outcomes in infants and young children, including negative cognitive and intellectual development outcomes; reduced academic potential; and increased risk of diarrhea, pneumonia, and anemia.^3,4,5,6^ Moreover, malnutrition can have intergenerational effects, leading to reduced physical strength, decreased work productivity, unemployment, and a higher risk of poverty.^7,8^

To address malnutrition, several global and regional initiatives have been implemented. The United Nations launched the Decade of Action on Nutrition, which aims to reduce the rate of stunting in children younger than 5 years by 40% by 2025,^9^ and the Sustainable Development Goals include a target to eradicate all forms of malnutrition by 2030.^10^ In Africa, the African Union developed the Africa Regional Nutrition Strategy to adopt the global targets of the global nutrition targets, and this was endorsed by the African Union member states.^11^ Furthermore, the Initiative for Food and Nutrition Security in Africa was launched with support from the government of Japan to establish a framework for nutritional security policies.^12^

Despite these efforts, recent reports^2^ indicated that malnutrition remains a major public health problem, with an estimated 149 million children younger than 5 years experiencing stunting. A pooled analysis from 35 low- and middle-income countries revealed a 39% pooled prevalence of childhood stunting.^13^ In Africa, no country has made substantial progress toward achieving the Sustainable Development Goals^1,2,3,4,10,11^ and global nutrition targets.^2,14,15^ The prevalence of stunting among children increased by 17% in Africa from 2000 to 2018, with the number of children experiencing stunting increasing from 50 million to 59 million, highlighting the urgent need for action in the region.^16^

Sub-Saharan Africa (SSA), despite its abundant cultural heritage and history, continues to be 1 of the most impoverished regions globally, facing high rates of malnutrition.^16,17^ Poverty,^18^ food insecurity,^19^ limited access to clean water and sanitation,^20^ and the widespread presence of diseases such as malaria and diarrhea^21,22^ contribute to the serious problem of malnutrition in the region. It is crucial to address the modifiable factors that contribute to malnutrition in children to substantially reduce its burden and to lead to improved health outcomes for families and communities.

Previous studies have identified numerous factors associated with increased risk of stunting in SSA. These factors included low levels of educational attainment among mothers,^23,24^ maternal unemployment,^24^ economic disadvantages,^23,24^ macronutrient or micronutrient deficiencies,^23,24^ episodes of diarrhea,^23^ low birth weight,^23^ lack of improved toilet facilities,^23^ and lack of access to clean water.^23^ Lack of access to media outlets,^25^ infrequent prenatal care visits,^25^ and home births^26^ were also positively associated with childhood stunting. While these studies are important and merit recognition, using relative measures of association alone to evaluate the consequences of factors may not be optimal for public health planning and resource allocation. The relative association between risk factors and outcomes, as indicated by odd ratios and relative risks (RRs), may not always correspond with their public health significance.^27,28^ For example, an association between a factor and outcome may have minimal public health importance if the factor is rare, while an association could have a greater impact if the factor is more common.

Using the population-attributable fraction (PAF) is a common approach that considers both the prevalence of the risk factor in the population and the association between the risk factor and the disease.^28,29,30^ This enables an estimate of the proportion of a specific outcome that can be potentially associated with an exposure in the study population as well as the potential reduction in the outcome if the exposure prevalence were hypothetically reduced to 0. Key assumptions also include that exposures are independent and associations between exposures and outcomes are unidirectional and constant through time.^31,32^ Despite these limitations, PAF estimates are a parsimonious way of quantifying risk that can supplement other approaches to identifying modifiable risk factors for policy intervention and prioritization. The PAF has been applied to various health outcomes such as stunting,^33^ postnatal care services,^34^ complementary feeding practices,^35^ maternal anemia,^27^ exclusive breastfeeding,^36^ and low birth weight.^37^ Our study focused on severe childhood stunting given its enduring associations with both physical and cognitive development^38,39,40^ and the need to target limited health care resources in SSA.^41^ We calculated the PAFs for common modifiable risk factors associated with stunting and severe stunting in children younger than 5 years using nationally representative surveys from 25 SSA countries.

## Methods

### Data Sources

This cross-sectional study used the latest Demographic and Health Surveys (DHS) data sets from 2014 to 2021 from 25 nations in the SSA region. The selection of these countries, including Angola, Benin, Burundi, Cameroon, Chad, Ethiopia, Ghana, Guinea, Kenya, Lesotho, Liberia, Madagascar, Malawi, Mali, Mauritania, Nigeria, Rwanda, Senegal, Sierra Leone, South Africa, Tanzania, The Gambia, Uganda, Zambia, and Zimbabwe, was based on the availability of recent (eg, DHS-VII or DHS-VIII) data sets that represent the current socioeconomic conditions of countries. The Monitoring and Evaluation to Assess and Use Results DHS were carried out by the respective country’s health ministry or governmental agencies, with support from Inner City Fund International, at regular intervals. The survey design and data are consistent, as they are based on standardized data collection methods and survey tools. The DHS gathers data on the demographics and health of individuals, encompassing topics such as maternal and child health, mortality, nutrition, and the social determinants of health.^42^ Ethical clearance was obtained for all DHS from the respective countries, whereas for this study, the requirement for informed consent was waived as publicly available data were used. This study followed the Strengthening the Reporting of Observational Studies in Epidemiology (STROBE) reporting guideline for cross-sectional studies.

### Design, Sampling, and Population

The DHS data were obtained using a 2-stage, stratified cluster sampling design that first included administrative units (eg, states and regions) as urban and rural strata. During the first stage, enumeration areas were randomly selected based on their size and a complete census of households was conducted in each selected enumeration area. In the second stage, a systematic random sampling approach was used to select a fixed number of households from each enumeration area with equal probability. For this study, we used the Children’s Data Set (Children’s Recode). From the Children’s Recode file, we extracted the dependent and independent variables for each nation and then appended and merged the DHS data from the 25 countries in SSA.^43^

Data were obtained from eligible women, who were defined as all females aged 15 to 49 years who either lived permanently or were visiting the households on the night before the survey. The present study was restricted to the youngest children aged 0 to 59 months who resided with the respondent across all 25 countries in the SSA region. Further details about the sampling design and questionnaire can be found in the country-specific Monitoring and Evaluation to Assess and Use Results DHS reports.

### Outcome Variables

The primary outcomes for this study were stunting and severe stunting, defined using the height-for-age *z* (HAZ) score from the World Health Organization child growth standards. Height and length were measured using a ShorrBoard (Weigh and Measure, LLC) in a lying position for children younger than 2 years and in a standing position for older children (aged 24-59 months). The stunting index (HAZ) was expressed in SD units from the median of the World Health Organization reference population for the child’s age. In accordance with DHS guidelines and prior studies,^43,44,45^ children were classified as having stunted or severely stunted growth when their HAZ score was below −2.0 SDs or −3.0 SDs, respectively.

### Modifiable Risk Factors

Modifiable risk factors were broadly grouped as child factors, maternal factors, and household factors, and the selection of these factors was based on evidence from previous studies^27,36,46^ and data availability. Child factors included recent diarrheal episodes, acute respiratory tract infections, and dairy product consumption. Maternal factors included body mass index, educational level, employment, antenatal care visits, and place of child’s birth (Table 1).

**Table 1.** Definitions of Modifiable Risk Factors Associated With Stunting and Severe Stunting Among Children Younger Than 5 Years in Sub-Saharan Africa

Factor	Definition
Child	
Recent diarrheal episodes	Passing of abnormal stools during the 2 weeks preceding the survey; grouped as 1, experienced diarrhea, or 2, did not experience diarrhea
ARI	Symptoms of cough and shortness of breath during the 2 weeks preceding the survey; grouped as 1, experienced ARI, or 2, did not experience ARI
Dairy products consumption	Children who consumed dairy products, including milk, infant formula, yogurt, and cheese, during the previous day
Maternal	
Body mass index^a^	Nutrition was grouped as 1, underweight; 2, normal weight; or 3, overweight or obesity
Educational level	Grouped as 1, no schooling; 2, primary education; or 3, secondary education or higher
Employment status	Grouped as not working or working
Frequency of antenatal care visits	Grouped as 1, none; 2, 1-3 visits; or 3, ≥4 visits
Place of birth	Grouped as home or health facility birth
Household	
Wealth index	Grouped as 1 lowest; 2, middle; or 3, highest
Source of drinking water and type of toilet facility	Grouped as 1, improved, or 2, not improved
Type of cooking fuel	Grouped as 1, cleaned, or 2, not cleaned

Abbreviation: ARI, acute respiratory tract infection.

^a^ Calculated as weight in kilograms divided by height in meters squared. Underweight is defined as less than 18.5; normal weight, 18.5 to 24.9; overweight or obese, 25.0 or greater.

Household factors included household wealth index, type of toilet system, source of drinking water, and type of cooking fuel (Table 1). The household wealth index was generated through the use of principal component analysis, which takes into account various factors such as the ownership of household amenities (eg, toilets, electricity, television, radio, refrigerator, and bicycle), the availability of a source of drinking water, and the type of flooring material used in the main house.^47^

### Potential Covariates

For this study, we considered potential covariates. These included the sex of the child (grouped as female or male), the child’s birth order (grouped as first child, 2 to <5 births, or ≥5 births), maternal age (grouped as 15-24 years, 25-34 years, or 35-49 years), and place of residence (grouped as rural or urban).

### Statistical Analysis

A 3-stage analytical strategy was used to examine modifiable risk factors associated with stunting and severe stunting among children younger than 5 years in 25 SSA countries. In stage 1, frequencies and percentages were calculated to describe the study population and examine the prevalence of stunting and severe stunting across study variables (including child, maternal, and household factors). Given the high level of heterogeneity in the sample size across countries, we used a random-effects meta-analysis to calculate the pooled prevalence of stunting and severe stunting. In stage 2, generalized linear latent and mixed models (GLLAMMs) using a log-binomial link were fitted to calculate RRs and 95% CIs for modifiable risk factors of stunting and severe stunting. The GLLAMM was selected to account for the hierarchical nature of the data (eg, children aged <5 years nested within geographic clusters), consistent with previously published studies.^36,48^ All regression models were adjusted for the variable place of residence while examining associations between modifiable risk factors and outcome variables.^36^

The final step involved the calculation of PAFs for modifiable risk factors that showed a significant association with stunting and severe stunting in the GLLAMM. The study used the RR as a measure of association between risk factors and outcomes to calculate the PAF. Our decision was influenced by several factors: (1) odds ratios can overestimate the risk in prevalent cases (≥10%); (2) the DHS use a random sampling technique, resulting in convergence of prevalence and incidence rates; (3) representative longitudinal studies or meta-analyses providing RRs for stunting or severe stunting were not found in SSA; and (4) as resources are limited, identifying priority-amenable risk factors is essential to tackle childhood malnutrition.

The PAF was calculated using the Levin formula, which assessed the proportion of stunting and severe stunting cases in SSA that could be avoided by eliminating modifiable risk factors among the population.^31^ Given that risk factors tend to occur together within individuals, adding up PAFs of each risk factor would result in an inflated estimate of their combined PAFs. Thus, we calculated a joint PAF across all risk factors.^36,49^ The formulas for the PAF and the joint PAF are presented in the eAppendix in Supplement 1.

To account for sampling weights, clustering, and stratification, the svy command in Stata, version 15.0 (StataCorp LLC) was used.^50^ In addition, denormalization of the sampling weight was applied to the combined data set, and a new weight at the population level was created to consider the unequal distribution of the population across SSA countries. This step was crucial because the size of the population in some countries (eg, Nigeria) was significantly greater than in others (eg, The Gambia), which had the potential to bias the results. The regression modeling was conducted using the GLLAMM package for Stata.^51^ The measure of association between modifiable risk factors and the outcome variables was reported as RRs, PAFs, and 95% CIs.

## Results

### Study Participants

Of the 145 900 children from 25 SSA countries, 49.4% were female and 50.6% were male; mean (SD) age was 29.4 (17.3) years. The study revealed that 15.1% of children had episodes of diarrhea (≥3 episodes per day) 2 weeks before the survey, and the majority (84.6%) did not consume dairy products in the past 24 hours. A total of 39.4% of mothers did not attain a formal level of education, and 66.6% were unemployed. A total of 55.0% of the children resided in households with an unimproved toilet system, and 63.9% were born at health facilities (eTable 1 in Supplement 1).

### Prevalence of Stunting and Severe Stunting

The overall prevalence of stunting was 30.6% (95% CI, 27.4%-34.1%). The highest prevalence of stunting was in Burundi (55.8%; 95% CI, 54.6%-57.1%) followed by Chad (39.6%; 95% CI, 38.7%-40.6%), while the lowest prevalence was in The Gambia (17.1%; 95% CI, 15.8%-18.3%) (eTable 2 in Supplement 1). The overall prevalence of severe stunting was 10.5% (95% CI, 8.8%-12.6%). The highest prevalence of severe stunting was in Burundi (24.8%; 95% CI, 23.7%-25.8%) followed by Chad (21.7%; 95% CI, 20.9%-22.5%), while the lowest prevalence was in The Gambia (3.5%; 95% CI, 2.9%-4.1%) (eTable 2 in Supplement 1).

### PAF for Stunting

The largest proportion of stunting cases was associated with a lack of dairy product consumption among children (PAF, 15.1%; 95% CI, 12.5%-17.5%) followed by mothers with no formal education (PAF, 11.2%; 95% CI, 9.7%-12.9%) and those with a primary educational level (PAF, 8.1%; 95% CI, 6.7%-9.4%). The PAFs for stunting associated with unclean cooking fuel and low-income households were also high (unclean cooking fuel: PAF, 9.5%; 95% CI, 6.5%-13.1%; households with limited wealth: PAF,  5.4%; 95% CI, 4.2%-6.7%) (Table 2). The combined PAFs showed that 40.7% (95% CI, 34.0%-47.2%) of cases of childhood stunting in SSA were associated with children lacking dairy products, mothers with a primary educational level, unclean cooking fuel, and low-income household.

**Table 2.** Population-Attributable Fractions for Risk Factors Associated With Stunting in Sub-Saharan Africa, 2014-2021

Factor	Proportion of exposure, % (95% CI)	Adjusted RR (95% CI)	PAF, % (95% CI)
Child			
Recent diarrheal episodes			
No	84.9 (84.6 to 85.2)	1 [Reference]	[Reference]
Yes	15.1 (14.8 to 15.5)	1.13 (1.11 to 1.16)	1.9 (1.6 to 2.4)
Dairy products consumption			
No	84.6 (84.2 to 85.0)	1.21 (1.17 to 1.25)	15.1 (12.5 to 17.5)
Yes	15.4 (15.0 to 15.8)	1 [Reference]	[Reference]
Maternal			
Body mass index^a^			
Normal weight	53.9 (53.3 to 54.4)	1 [Reference]	[Reference]
Underweight	7.8 (7.5 to 8.1)	1.13 (1.10 to 1.16)	1.0 (0.7 to 1.3)
Overweight or obesity	38.2 (37.5 to 38.9)	0.80 (0.78 to 0.82)	−8.3 (−0.9 to −7.5)
Educational level			
No formal education	39.4 (38.5 to 40.2)	1.32 (1.28 to 1.37)	11.2 (9.7 to 13.0)
Primary education	35.0 (34.4 to 35.6)	1.25 (1.21 to 1.29)	8.0 (6.7 to 9. 4)
Secondary or higher	25.6 (25.0 to 26.2)	1 [Reference]	[Reference]
Antenatal care			
None	11.1 (10.5 to 11.6)	1.18 (1.15 to 1.22)	1.9 (1.6 to 2.5)
1-3 Visits	34.1 (33.6 to 34.6)	1.10 (1.07 to 1.12)	3.3 (2.3 to 4.0)
≥4 Visits	54.9 (54.2 to 55.5)	1 [Reference]	[Reference]
Place of birth			
Home	36.1 (35.2 to 37.0)	1.06 (1.03 to 1.08)	2.1 (1.0 to 2.8)
Health facility	63.9 (63.0 to 64.8)	1 [Reference]	[Reference]
Household			
Wealth index			
Lowest	44.1 (43.3 to 45.0)	1.13 (1.10 to 1.16)	5.4 (4.2 to 6.7)
Middle	20.0 (19.5 to 20.4)	1.10 (1.07 to 1.12)	2.0 (1.4 to 2.4)
Highest	35.9 (35.1 to 36.8)	1 [Reference]	[Reference]
Type of toilet system			
Not improved	55.0 (54.1 to 55.9)	1.02 (1.00 to 1.04)	1.1 (0.0 to 2.2)
Improved	45.6 (44.7 to 46.5)	1 [Reference]	[Reference]
Type of cooking fuel			
Not cleaned	87.8 (87.2 to 88.4)	1.12 (1.08 to 1.17)	9.5 (6.5 to 13.1)
Cleaned	12.2 (11.6 to 12.7)	1 [Reference]	[Reference]

Abbreviations: PAF, population-attributable fraction; RR, relative risk.

^a^ Calculated as weight in kilograms divided by height in meters squared. Underweight is defined as less than 18.5; normal weight, 18.5 to 24.9; overweight or obese, 25.0 or greater.

### PAF for Severe Stunting

The highest proportion of severe stunting was associated with mothers not having a formal education (PAF, 21.9%; 95% CI, 19.0%-24.8%) followed by children not consuming dairy products (PAF, 20.8%; 95% CI, 16.8%-24.9%). The PAFs for severe stunting associated with unclean cooking fuel and home birth were also high (unclean cooking fuel: PAF, 9.5%; 95% CI, 2.6%-16.3%; home birth: PAF, 8.3%; 95% CI, 6.3%-10.0%). Of cases of severe stunting, 5.8% (95% CI: 3.3, 8.0) were associated with low-income household (Table 3). The combined PAFs showed that 51.6% (95% CI, 40.5%-60.9%) of cases of severe childhood stunting in SSA were associated with no formal education among mothers, children lacking dairy products, unclean cooking fuel, home birth, and low-income household.

**Table 3.** Population-Attributable Fractions for Risk Factors Associated With Severe Stunting in Sub-Saharan Africa, 2014-2021

Factor	Proportion of exposure, % (95% CI)	Adjusted RR (95% CI)	PAF, % (95% CI)
Child			
Recent diarrheal episodes			
No	84.9 (84.6 to 85.2)	1 [Reference]	[Reference]
Yes	15.1 (14.8 to 15.5)	1.19 (1.14 to 1.24)	2.8 (2.0 to 3.6)
Dairy products consumption			
No	84.6 (84.2 to 85.0)	1.31 (1.24 to 1.39)	20.8 (16.8 to 24.9)
Yes	15.4 (15.0 to 15.8)	1 [Reference]	[Reference]
Maternal			
Body mass index^a^			
Normal weight	53.9 (53.3 to 54.4)	1 [Reference]	[Reference]
Underweight	7.8 (7.5 to 8.1)	1.21 (1.15 to 1.27)	1.6 (1.1 to 2.1)
Overweight or obesity	38.2 (37.5 to 38.9)	0.73 (0.70 to 0.77)	−11.5 (−12.7 to −9.8)
Educational level			
No formal education	39.4 (38.5 to 40.2)	1.71 (1.61 to 1.82)	21.9 (19.0 to 24.8)
Primary education	35.0 (34.4 to 35.6)	1.34 (1.26 to 1.43)	10.6 (8.2 to 13.3)
Secondary or higher	25.6 (25.0 to 26.2)	1 [Reference]	[Reference]
Antenatal care			
None	11.1 (10.5 to 11.6)	1.41 (1.33 to 1.48)	4.4 (3.4 to 5.3)
1-3 Visits	34.1 (33.6 to 34.6)	1.14 (1.09 to 1.19)	4.6 (2.9 to 6.2)
≥4 Visits	54.9 (54.2 to 55.5)	1 [Reference]	[Reference]
Place of birth			
Home	36.1 (35.2 to 37.0)	1.25 (1.19 to 1.30)	8.3 (6.3 to 10.0)
Health facility	63.9 (63.0 to 64.8)	1 [Reference]	[Reference]
Household			
Wealth index			
Lowest	44.1 (43.3 to 45.0)	1.14 (1.08 to 1.20)	5.8 (3.4 to 8.0)
Middle	20.0 (19.5 to 20.4)	1.09 (1.02 to 1.15)	1.8 (0.4 to 3.0)
Highest	35.9 (35.1 to 36.8)	1 [Reference]	[Reference]
Type of toilet system			
Not improved	55.0 (54.1 to 55.9)	1.07 (1.02 to 1.11)	3.7 (1.1 to 5.8)
Improved	45.6 (44.7 to 46.5)	1 [Reference]	[Reference]
Type of cooking fuel			
Not cleaned	87.8 (87.2 to 88.4)	1.12 (1.03 to 1.22)	9.5 (2.6 to 16.3)
Cleaned	12.2 (11.6 to 12.7)	1 [Reference]	[Reference]

Abbreviations: PAF, population-attributable fraction; RR, relative risk.

^a^ Calculated as weight in kilograms divided by height in meters squared. Underweight is defined as less than 18.5; normal weight, 18.5 to 24.9; overweight or obese, 25.0 or greater.

## Discussion

This study investigated the PAFs of stunting and severe stunting among children younger than 5 years using nationally representative surveys from 25 SSA countries. The greatest PAFs of severe stunting were observed among mothers lacking a formal education, children lacking dairy product consumption, unclean cooking fuel, home birth, and low-income household. In combination, these 5 modifiable risk factors were associated with 51.6% of cases of severe childhood stunting in SSA.

In the post–COVID-19 era, the global health community currently faces important challenges in meeting the estimated cost of $97 billion required to achieve the global nutrition targets by 2030.^52^ While this cost estimate does not include additional costs related to the management of COVID-19 and the health-related challenges from the Ukraine conflict, the attainment of the global nutrition targets appears to be unfeasible.^52^ In SSA countries, childhood malnutrition is an important public health concern, with varied levels of childhood undernutrition between and within countries.^15^ The improvements in global childhood nutrition will require investments that are directed toward the most effective interventions that maximize existing resources and capabilities.^52,53^ Our study underscores the need to prioritize resources based on the order of importance of the modifiable risk factors identified to be associated with stunting and severe stunting in children, namely a lack of a mother’s formal education (21.9%), a lack of dairy product consumption among children (20.8%), unclean cooking fuel (9.5%), home birth (8.3%), and poverty (5.8%). These findings can inform funding, public health practice, and policy priorities to address severe childhood stunting.

Health care workers will be vital for the delivery of future evidence-based health interventions to rural households. Pregnant individuals should be educated on an appropriate diet during pregnancy; the advantage of health care facility births compared with home births; culturally appropriate weaning foods; financial initiatives (eg, microenterprises); and suitable foods for their growing families. In sum, cost-effective choices should be made within rural homes if childhood malnutrition is to be addressed.

### Strengths and Limitations

This study has strengths. The estimation of PAFs for childhood stunting may, for the first time to our knowledge, aid in prioritizing resources toward reducing chronic childhood malnutrition within SSA. Additionally, the use of nationally representative DHS data sets in the study enhanced the generalizability of our findings to the region. The modifiable risk factors examined in the study also had general child health policy implications and potential associations with other early life and child health outcomes.

This study also has limitations. First, the study’s reliance on cross-sectional data made it difficult to clearly establish a temporal relationship between some putative exposures and the outcome. However, the modifiable risk factors identified align with previous studies conducted in SSA countries.^23,24,25^ Second, our findings may have been limited due to the absence of unmeasured modifiable risk factors, such as household food security and sociocultural factors, in the DHS data sets used. Nevertheless, we still examined the impact of household socioeconomic status, which can serve as a proxy for household food security and sociocultural practices. Third, the possibility of recall bias for certain modifiable risk factors, such as dairy product consumption, may have affected some of the results. Nevertheless, we attempted to address this bias by restricting the study population to the youngest child. Lastly, misclassification bias, including classifying common colds as acute respiratory tract infections or minor changes in bowel habits as diarrhea, as well as incorrectly categorizing household characteristics like cooking fuel and sanitation facilities, may have influenced the results.

There are also important underlying assumptions of PAF estimates, namely that there is a causal relationship between a given exposure and stunting. Population-attributable fraction estimates assume that the estimates of RR and prevalence are not affected by bias or confounding, that exposure variables are independent, and that associations are linear and constant over time.^31,32,54,55^ These are unlikely assumptions given the likely complex interactions among the socioeconomic, cultural, health service, maternal, and child factors that are associated with stunting. However, PAFs provide a straightforward and intuitive metric that can supplement other priority-setting approaches when identifying modifiable risk factors for policy intervention.

## Conclusions

In this cross-sectional study of 145 900 children from 25 SSA countries, we identified 5 modifiable risk factors that were associated with 51.6% of severe childhood stunting. Given the current global economy, we believe that these factors should be a priority for policy makers when considering future child health interventions to address chronic malnutrition. Health care workers will be vital for the delivery of such interventions to pregnant individuals in rural Africa.

## Notes

Supplement 1. **eTable 1.** Characteristics of Study Participants for Stunting and Severe Stunting Among Children Under Five in Sub-Saharan Africa Countries, 2014-2021 (145,900) **eTable 2.** Prevalence of Stunting and Severe Stunting Among Children in Sub-Saharan Africa Countries, 2014-2021 **eAppendix.** Formulas for the Population Attributable Fraction and the Joint Population Attributable Fraction

Supplement 2.
Data Sharing Statement

## References

[1] Black RE, Victora CG, Walker SP, ; Maternal and Child Nutrition Study Group. Maternal and child undernutrition and overweight in low-income and middle-income countries. Lancet. 2013;382(9890):427-451. doi:10.1016/S0140-6736(13)60937-X 23746772

[2] Development Initiatives. 2020 Global Nutrition Report: action on equity to end malnutrition. Accessed August 24, 2022. https://globalnutritionreport.org/reports/2020-global-nutrition-report/

[3] Kirolos A, Goyheneix M, Kalmus Eliasz M, . Neurodevelopmental, cognitive, behavioural and mental health impairments following childhood malnutrition: a systematic review. BMJ Glob Health. 2022;7(7):e009330. doi:10.1136/bmjgh-2022-009330 35793839 PMC9260807

[4] Pizzol D, Tudor F, Racalbuto V, Bertoldo A, Veronese N, Smith L. Systematic review and meta-analysis found that malnutrition was associated with poor cognitive development. Acta Paediatr. 2021;110(10):2704-2710. doi:10.1111/apa.15964 34077582

[5] Kirolos A, Blacow RM, Parajuli A, . The impact of childhood malnutrition on mortality from pneumonia: a systematic review and network meta-analysis. BMJ Glob Health. 2021;6(11):e007411. doi:10.1136/bmjgh-2021-007411 34848440 PMC8634228

[6] Brennhofer S, Reifsnider E, Bruening M. Malnutrition coupled with diarrheal and respiratory infections among children in Asia: a systematic review. Public Health Nurs. 2017;34(4):401-409. doi:10.1111/phn.12273 27354205

[7] Siddiqui F, Salam RA, Lassi ZS, Das JK. The intertwined relationship between malnutrition and poverty. Front Public Health. 2020;8:453. doi:10.3389/fpubh.2020.00453 PMC7485412 32984245

[8] Hoddinott J, Maluccio JA, Behrman JR, Flores R, Martorell R. Effect of a nutrition intervention during early childhood on economic productivity in Guatemalan adults. Lancet. 2008;371(9610):411-416. doi:10.1016/S0140-6736(08)60205-6 18242415

[9] World Health Organization. United Nations Decade of Action on Nutrition (2016-2025). May 28, 2016. Accessed December 19, 2022. https://apps.who.int/iris/bitstream/handle/10665/252788/A69_R8-en.pdf?sequence=1&isAllowed=y

[10] United Nations. Transforming our world: the 2030 agenda for sustainable development. Accessed February 26, 2022. https://sustainabledevelopment.un.org/content/documents/21252030%20Agenda%20for%20Sustainable%20Development%20web.pdf

[11] African Union Southern Africa Regional Office. Africa regional nutrition strategy 2015–2025. September 5, 2022. Accessed August 29, 2022. https://saro.au.int/en/documents/2022-09-05/africa-regional-nutrition-strategy-2015-2025

[12] Initiative for Food and Nutrition Security in Africa. Home age. Accessed October 1, 2021. https://ifna.africa

[13] Li Z, Kim R, Vollmer S, Subramanian SV. Factors associated with child stunting, wasting, and underweight in 35 low- and middle-income countries. JAMA Netw Open. 2020;3(4):e203386. doi:10.1001/jamanetworkopen.2020.3386 32320037 PMC7177203

[14] World Health Organization. Global targets 2025: global targets tracking tool. Accessed March 2, 2023. https://www.who.int/tools/global-targets-tracking-tool

[15] United Nations Children’s Fund, World Health Organization, World Bank Group. Levels and trends in child malnutrition. Accessed August 29, 2022. https://www.who.int/publications/i/item/9789240025257

[16] United Nations Children’s Fund. The state of the world’s children 2019. Accessed February 27, 2022. https://www.unicef.org/reports/state-of-worlds-children-2019

[17] Otekunrin OA, Otekunrin OA, Sawicka B, Ayinde IA. Three decades of fighting against hunger in Africa: progress, challenges and opportunities. World Nutrition. 2020;11(3):86-111. doi:10.26596/wn.202011386-111

[18] Adeyeye SAO, Ashaolu TJ, Bolaji OT, Abegunde TA, Omoyajowo AO. Africa and the nexus of poverty, malnutrition and diseases. Crit Rev Food Sci Nutr. 2023;63(5):641-656. doi:10.1080/10408398.2021.1952160 34259104

[19] Gassara G, Chen J. Household food insecurity, dietary diversity, and stunting in sub-Saharan Africa: a systematic review. Nutrients. 2021;13(12):4401. doi:10.3390/nu13124401 34959953 PMC8707760

[20] Momberg DJ, Ngandu BC, Voth-Gaeddert LE, . Water, sanitation and hygiene (WASH) in sub-Saharan Africa and associations with undernutrition, and governance in children under five years of age: a systematic review. J Dev Orig Health Dis. 2021;12(1):6-33. doi:10.1017/S2040174419000898 31902390

[21] Ahmed KY, Agho KE, Page A, Arora A, Ogbo FA; Global Maternal and Child Health Research Collaboration GloMACH. Mapping geographical differences and examining the determinants of childhood stunting in Ethiopia: a Bayesian geostatistical analysis. Nutrients. 2021;13(6):2104. doi:10.3390/nu13062104 34205375 PMC8234472

[22] Das D, Grais RF, Okiro EA, . Complex interactions between malaria and malnutrition: a systematic literature review. BMC Med. 2018;16(1):186. doi:10.1186/s12916-018-1177-5 30371344 PMC6205776

[23] Takele BA, Gezie LD, Alamneh TS. Pooled prevalence of stunting and associated factors among children aged 6-59 months in sub-Saharan Africa countries: a Bayesian multilevel approach. PLoS One. 2022;17(10):e0275889. doi:10.1371/journal.pone.0275889 36228030 PMC9560624

[24] Quamme SH, Iversen PO. Prevalence of child stunting in sub-Saharan Africa and its risk factors. Clin Nutr Open Sci. 2022;42:49-61. doi:10.1016/j.nutos.2022.01.009

[25] Adedokun ST, Yaya S. Factors associated with adverse nutritional status of children in sub-Saharan Africa: evidence from the Demographic and Health Surveys from 31 countries. Matern Child Nutr. 2021;17(3):e13198. doi:10.1111/mcn.13198 33960678 PMC8189196

[26] Amadu I, Seidu A-A, Duku E, . Risk factors associated with the coexistence of stunting, underweight, and wasting in children under 5 from 31 sub-Saharan African countries. BMJ Open. 2021;11(12):e052267. doi:10.1136/bmjopen-2021-052267 34930735 PMC8689177

[27] Kibret KT, Chojenta C, D’Arcy E, Loxton D. Population attributable fraction estimates for factors associated with different types of anaemia among women in Ethiopia: multilevel multinomial analysis. Public Health Nutr. 2021;24(13):4166-4176. doi:10.1017/S1368980020003109 32907664 PMC10195278

[28] Newson R. PUNAF: Stata module to compute population attributable fractions for cohort studies. Statistical Software Components S457193. Boston College Dept of Economics. 2010. Revised September 4, 2015. Accessed March 3, 2023. https://ideas.repec.org/c/boc/bocode/s457193.html

[29] Newson RB. Attributable and unattributable risks and fractions and other scenario comparisons. Stata J. 2013;13(4):672-698. doi:10.1177/1536867X1301300402

[30] Poole C. A history of the population attributable fraction and related measures. Ann Epidemiol. 2015;25(3):147-154. doi:10.1016/j.annepidem.2014.11.015 25721747

[31] Eide GE. Attributable fractions for partitioning risk and evaluating disease prevention: a practical guide. Clin Respir J. 2008;2(suppl 1):92-103. doi:10.1111/j.1752-699X.2008.00091.x 20298357

[32] Page A, Atkinson JA, Heffernan M, . Static metrics of impact for a dynamic problem: the need for smarter tools to guide suicide prevention planning and investment. Aust N Z J Psychiatry. 2018;52(7):660-667. doi:10.1177/0004867417752866 29359569

[33] Corsi DJ, Mejía-Guevara I, Subramanian SV. Risk factors for chronic undernutrition among children in India: estimating relative importance, population attributable risk and fractions. Soc Sci Med. 2016;157:165-185. doi:10.1016/j.socscimed.2015.11.014 26625852

[34] Agho KE, Ezeh OK, Issaka AI, Enoma AI, Baines S, Renzaho AM. Population attributable risk estimates for factors associated with non-use of postnatal care services among women in Nigeria. BMJ Open. 2016;6(7):e010493. doi:10.1136/bmjopen-2015-010493 27371552 PMC4947753

[35] Issaka AI, Agho KE, Ezeh OK, Renzaho AM. Population-attributable risk estimates for factors associated with inappropriate complementary feeding practices in The Gambia. Public Health Nutr. 2017;20(17):3135-3144. doi:10.1017/S1368980017002014 28847321 PMC10261515

[36] Ogbo FA, Page A, Idoko J, Agho KE. Population attributable risk of key modifiable risk factors associated with non-exclusive breastfeeding in Nigeria. BMC Public Health. 2018;18(1):247. doi:10.1186/s12889-018-5145-y 29439701 PMC5812198

[37] Bada HS, Das A, Bauer CR, . Low birth weight and preterm births: etiologic fraction attributable to prenatal drug exposure. J Perinatol. 2005;25(10):631-637. doi:10.1038/sj.jp.7211378 16107872

[38] Olofin I, McDonald CM, Ezzati M, ; Nutrition Impact Model Study (anthropometry cohort pooling). Associations of suboptimal growth with all-cause and cause-specific mortality in children under five years: a pooled analysis of ten prospective studies. PLoS One. 2013;8(5):e64636. doi:10.1371/journal.pone.0064636 23734210 PMC3667136

[39] Grey K, Gonzales GB, Abera M, . Severe malnutrition or famine exposure in childhood and cardiometabolic non-communicable disease later in life: a systematic review. BMJ Glob Health. 2021;6(3):e003161. doi:10.1136/bmjgh-2020-003161 33692144 PMC7949429

[40] Mendez MA, Adair LS. Severity and timing of stunting in the first two years of life affect performance on cognitive tests in late childhood. J Nutr. 1999;129(8):1555-1562. doi:10.1093/jn/129.8.1555 10419990

[41] Asante A, Wasike WSK, Ataguba JE. Health financing in sub-Saharan Africa: from analytical frameworks to empirical evaluation. Appl Health Econ Health Policy. 2020;18(6):743-746. doi:10.1007/s40258-020-00618-0 33145665 PMC7609366

[42] Corsi DJ, Neuman M, Finlay JE, Subramanian SV. Demographic and Health Surveys: a profile. Int J Epidemiol. 2012;41(6):1602-1613. doi:10.1093/ije/dys184 23148108

[43] US Agency for International Development. Guide to DHS statistics: DHS-7—the Demographic and Health Surveys Program. 2018. Accessed February 26, 2022. https://dhsprogram.com/pubs/pdf/DHSG1/Guide_to_DHS_Statistics_DHS-7.pdf

[44] Akombi BJ, Agho KE, Hall JJ, Merom D, Astell-Burt T, Renzaho AMN. Stunting and severe stunting among children under-5 years in Nigeria: a multilevel analysis. BMC Pediatr. 2017;17(1):15. doi:10.1186/s12887-016-0770-z 28086835 PMC5237247

[45] Dhami MV, Ogbo FA, Osuagwu UL, Ugboma Z, Agho KE. Stunting and severe stunting among infants in India: the role of delayed introduction of complementary foods and community and household factors. Glob Health Action. 2019;12(1):1638020. doi:10.1080/16549716.2019.1638020 31333077 PMC7011976

[46] Lee M, Whitsel E, Avery C, . Variation in population attributable fraction of dementia associated with potentially modifiable risk factors by race and ethnicity in the US. JAMA Netw Open. 2022;5(7):e2219672. doi:10.1001/jamanetworkopen.2022.19672 35793088 PMC9260480

[47] Vyas S, Kumaranayake L. Constructing socio-economic status indices: how to use principal components analysis. Health Policy Plan. 2006;21(6):459-468. doi:10.1093/heapol/czl029 17030551

[48] Kaforau LS, Tessema GA, Jancey J, Bugoro H, Pereira G. Prevalence and risk factors associated with under-five mortality in the Solomon Islands: an investigation from the 2015 Solomon Islands demographic and health survey data. Lancet Reg Health West Pac. 2023;33:100691. doi:10.1016/j.lanwpc.2023.100691 37181533 PMC10166993

[49] Wilson LF, Page AN, Dunn NAM, Pandeya N, Protani MM, Taylor RJ. Population attributable risk of modifiable risk factors associated with invasive breast cancer in women aged 45-69 years in Queensland, Australia. Maturitas. 2013;76(4):370-376. doi:10.1016/j.maturitas.2013.09.002 24113278

[50] StataCorp LLC. Stata Survey Data Reference Manual: Release 15. Stata Press; 2017.

[51] Rabe-Hesketh S. GLLAMM: Stata Program to Fit Generalised Linear Latent and Mixed Models. Boston College Dept of Economics; 2000.

[52] Breaking the Cycle of Chronic Child Malnutrition in Sub-Saharan Africa: Economist Impact Report. Abbott; 2023:1-29.

[53] Scott N, Delport D, Hainsworth S, . Ending malnutrition in all its forms requires scaling up proven nutrition interventions and much more: a 129-country analysis. BMC Med. 2020;18(1):356. doi:10.1186/s12916-020-01786-5 33183301 PMC7661178

[54] Rockhill B, Newman B, Weinberg C. Use and misuse of population attributable fractions. Am J Public Health. 1998;88(1):15-19. doi:10.2105/AJPH.88.1.15 9584027 PMC1508384

[55] Sterman JD. Learning from evidence in a complex world. Am J Public Health. 2006;96(3):505-514. doi:10.2105/AJPH.2005.066043 16449579 PMC1470513

