# Supplementary material for: Population-Modifiable Risk Factors Associated With Childhood Stunting in Sub-Saharan Africa
Source: JAMA Netw Open. 2023 Oct 18;6(10):e2338321. doi: 10.1001/jamanetworkopen.2023.38321 (PMC10585405; doi:10.1001/jamanetworkopen.2023.38321)
Supplement: Supplement 1. — eTable 1. Characteristics of Study Participants for Stunting and Severe Stunting Among Children Under Five in Sub-Saharan Africa Countries, 2014-2021 (145,900) eTable 2. Prevalence of Stunting and Severe Stunting Among Children in Sub-Saharan Africa Countries, 2014-2021 eAppendix. Formulas for the Population Attributable Fraction and the Joint Population Attributable Fraction [file jamanetwopen-e2338321-s001.pdf]

Supplemental Online Content

Ahmed KY, Dadi AF, Ogbo FA, et al. Population-modifiable risk factors associated with childhood stunting in sub-Saharan Africa. *JAMA Netw Open*. 2023;6(10):e2338321. doi:10.1001/jamanetworkopen.2023.38321

eTable 1. Characteristics of Study Participants for Stunting and Severe Stunting Among Children Under Five in Sub-Saharan Africa Countries, 2014-2021 (145,900)
eTable 2. Prevalence of Stunting and Severe Stunting Among Children in Sub-Saharan Africa Countries, 2014-2021
eAppendix. Formulas for the Population Attributable Fraction and the Joint Population Attributable Fraction

This supplemental material has been provided by the authors to give readers additional information about their work.

© 2023 Ahmed KY et al. *JAMA Network Open*.

eTable 1. Characteristics of Study Participants for Stunting and Severe Stunting Among Children Under Five in Sub-Saharan Africa Countries, 2014-2021 (145,900)

Variables	Total population	Stunting		Severe stunting	
	n (%)	Yes, n (%)	No, n (%)	Yes, n (%)	No, n (%)
**Child factors**					
Recent diarrhoeal episodes					
No	123626 (84.9)	38203 (82.9)	85423 (85.8)	14213 (81.8)	109413 (85.3)
Yes	22041 (15.1)	7885 (17.1)	14156 (14.2)	3158 (18.2)	18883 (14.7)
Acute respiratory tract infections					
No	138769 (95.1)	43729 (94.7)	95040 (95.3)	16410 (94.9)	122360 (95.2)
Yes	7132 (4.9)	2436 (5.3)	4695 (4.7)	995 (5.1)	6137 (4.8)
Dairy products consumption					
No	123439 (84.6)	40329 (87.4)	83110 (83.3)	15321 (88.0)	108118 (84.1)
Yes	22462 (15.4)	5837 (12.6)	16625 (16.7)	2083 (12.0)	20379 (15.9)
**Maternal factors**					
Maternal BMI					
Normal weight	75932 (53.9)	27237 (60.2)	48695 (50.8)	10671 (62.2)	65261 (52.8)
Underweight	11017 (7.8)	4682 (10.3)	6335 (6.6)	2092 (12.2)	8925 (7.2)
Overweight/obesity	53851 (38.2)	13348 (29.5)	40504 (42.6)	4398 (25.6)	49453 (40.0)
Maternal education					
No formal education	57460 (39.4)	21322 (46.2)	36139 (36.2)	9279 (53.3)	48182 (37.5)
Primary education	51090 (35.0)	16964 (36.7)	34125 (34.2)	5883 (33.8)	45207 (35.2)
Secondary or higher	37345 (25.6)	7880 (17.1)	29466 (29.5)	2243 (12.9)	35102 (27.3)
Maternal employment					
Not working	91251 (66.6)	29827 (68.0)	61424 (66.0)	11013 (65.9)	80238 (66.7)
Working	45666 (33.4)	14018 (32.0)	31648 (34.0)	5691 (34.1)	39976 (33.3)
Antenatal care					
None	11383 (11.1)	4633 (15.1)	6750 (9.3)	2266 (20.4)	9117 (9.9)
1-3 visits	35084 (34.1)	11259 (36.7)	23825 (33.0)	4041 (36.4)	31042 (33.8)
4+ visits	56448 (54.8)	14749 (48.1)	41699 (57.7)	4806 (43.2)	51642 (56.2)
Place of birth					
Home	52659 (36.1)	20399 (44.2)	32261 (32.4)	8958 (51.5)	43701 (34.0)
Health facility	93162 (63.9)	25743 (55.8)	67419 (67.6)	8435 (48.5)	84727 (66.0)
**Household factors**					
Household wealth					
Poor	64358 (44.1)	24577 (53.2)	39780 (40.0)	9955 (57.2)	54402 (42.3)
Middle	29157 (20.0)	9450 (20.5)	19707 (19.7)	3442 (19.8)	25715 (20.0)
Rich	52386 (35.9)	12138 (26.3)	40248 (40.3)	4007 (23.0)	48379 (37.7)
Type of toilet system					
Not improved	78242 (55.0)	28227 (63.0)	50015 (51.3)	11487 (67.9)	66755 (53.2)
Improved	64098 (45.0)	16576 (37.0)	47522 (48.7)	5434 (32.1)	58664 (46.8)
Source of drinking water					
Not Protected	66589 (45.6)	23166 (50.2)	43423 (43.5)	8914 (51.2)	57675 (44.9)
Protected	79312 (54.4)	23000 (49.8)	56312 (56.5)	8490 (48.8)	70822 (55.1)
Type of cooking fuel					
Not cleaned	127953 (87.8)	42825 (92.8)	85129 (85.5)	16377 (94.1)	111577 (87.0)
Cleaned	17742 (12.2)	3315 (7.2)	14427 (14.5)	1019 (5.9)	16723 (13.0)

© 2023 Ahmed KY et al. *JAMA Network Open*.

eTable 2. Prevalence of Stunting and Severe Stunting Among Children in Sub-Saharan Africa Countries, 2014-2021

Countries	Stunting			Severe stunting		
	Prevalence	Upper interval	Lower interval	Prevalence	Upper interval	Lower interval
Angola, DHS 2015-16	37.0	35.8	38.3	14.6	13.7	15.6
Benin, DHS 2017-18	31.6	30.8	32.5	10.5	9.9	11.0
Burundi, DHS 2016-17	55.8	54.6	57.1	24.8	23.7	25.8
Cameroon, DHS 2018	29.2	27.9	30.6	13.7	12.7	14.7
Chad, DHS 2014-15	39.6	38.7	40.6	21.7	20.9	22.5
Ethiopia, DHS 2015-16	38.4	37.4	39.4	17.4	16.6	18.1
Gambia, DHS 2019-20	17.1	15.8	18.3	3.5	2.9	4.1
Ghana, DHS 2014	17.9	16.5	19.5	4.8	4.0	5.7
Guinea, DHS 2018	31.0	29.5	32.6	13.7	12.6	14.9
Kenya, DHS 2014	25.8	25.1	26.5	7.8	7.4	8.1
Lesotho, DHS 2014	32.4	29.9	35.1	10.5	8.9	12.3
Liberia, DHS 2019-20	28.8	26.9	30.8	8.8	7.6	10.1
Madagascar, DHS 2021	39.3	38.1	40.6	12.5	11.7	13.5
Malawi, DHS 2015-16	36.6	35.2	37.9	10.5	9.7	11.4
Mali, DHS 2018	26.8	25.9	27.7	10.1	9.4	10.7
Mauritania, DHS 2019-21	25.4	24.5	26.3	8.3	7.8	8.9
Nigeria, DHS 2018	36.6	35.7	37.5	16.9	16.2	17.6
Rwanda, DHS 2019-20	33.2	31.7	34.7	9.0	8.1	10.0
Senegal, DHS 2019	17.7	16.6	18.7	4.8	4.2	5.4
Sierra Leone, DHS 2019	29.0	27.6	30.5	10.3	9.4	11.3
South Africa, DHS 2016	26.6	23.9	29.3	10.1	8.3	12.0
Tanzania, DHS 2015-16	34.1	33.1	35.1	11.4	10.8	12.1
Uganda, DHS 2016	28.2	26.8	29.5	8.9	8.1	9.8
Zambia, DHS 2018	34.7	33.7	35.7	11.7	11.0	12.4
Zimbabwe, DHS 2014-15	26.3	25.1	27.6	8.2	7.4	8.9
**Pooled 25 SSA countries**	**30.6**	**27.4**	**34.1**	**10.5**	**8.8**	**12.6**

© 2023 Ahmed KY et al. *JAMA Network Open*.

eAppendix. Formulas for the Population Attributable Fraction and the Joint Population Attributable Fraction

PAF was calculated using Levin's formula, which determined the proportion of stunting and severe stunting cases in SSA that could be avoided by eliminating modifiable risk factors among the population [1]. PAF was calculated using the following formula:

$$PAF = (P * (RR - 1)) / (1 + (P * (RR - 1)))$$

Where p is the prevalence of the risk factor in the population, and RR is the adjusted RRs of stunting and severe stunting associated with the modifiable risk factor[1, 2].

Because risk factors tend to occur together within individuals, adding up the PAFs of each risk factor would result in an inflated estimate of their combined PAFs. Thus, we calculated a joint PAF across all risk factors using the formula[3, 4]:

$$PAF\ (combined) = 1 - \prod_{r=1}^{R} (1 - PAFr)$$

Where *r* represents each exposure variable. The assumption that exposures are independent and uncorrelated has been diminished with the use of relative risks that have been adjusted for potential confounders.

1. Eide GE. Attributable fractions for partitioning risk and evaluating disease prevention: a practical guide. *Clin Respir J* 2008;2 Suppl 1:92-103. doi: 10.1111/j.1752-699X.2008.00091.x
2. Lin C-K, Chen S-T. Estimation and application of population attributable fraction in ecological studies. *Environmental Health* 2019;18(1):52. doi: 10.1186/s12940-019-0492-4
3. Ogbo FA, Page A, Idoko J, et al. Population attributable risk of key modifiable risk factors associated with non-exclusive breastfeeding in Nigeria. *BMC public health* 2018;18(1):247. doi: 10.1186/s12889-018-5145-y [published Online First: 20180213]
4. Wilson LF, Page AN, Dunn NAM, et al. Population attributable risk of modifiable risk factors associated with invasive breast cancer in women aged 45–69 years in Queensland, Australia. *Maturitas* 2013;76(4):370-76. doi: https://doi.org/10.1016/j.maturitas.2013.09.002

© 2023 Ahmed KY et al. *JAMA Network Open*.
