# Supplementary material for: Population-Modifiable Risk Factors Associated With Childhood Stunting in Sub-Saharan Africa
Source: JAMA Netw Open. 2023 Oct 18;6(10):e2338321. doi: 10.1001/jamanetworkopen.2023.38321 (PMC10585405; doi:10.1001/jamanetworkopen.2023.38321)
Supplement: Supplement 2. — Data Sharing Statement [file jamanetwopen-e2338321-s002.pdf]

Data Sharing Statement

Ahmed. Population-Modifiable Risk Factors Associated With Childhood Stunting in Sub-Saharan Africa. *JAMA Netw Open*. Published October 18, 2023.
doi:10.1001/jamanetworkopen.2023.38321

Data
Data available: No

Additional Information
Explanation for why data not available: The analysis was based on the Demographic Health Survey. Information on the data and content can be accessed at
https://dhsprogram.com/data/available-datasets.cfm
